# Motor Point Stimulation in Spinal Paired Associative Stimulation can Facilitate Spinal Cord Excitability

**DOI:** 10.3389/fnhum.2020.593806

**Published:** 2020-11-27

**Authors:** Kai Lon Fok, Naotsugu Kaneko, Atsushi Sasaki, Kento Nakagawa, Kimitaka Nakazawa, Kei Masani

**Affiliations:** ^1^Institute of Biomedical Engineering, University of Toronto, Toronto, ON, Canada; ^2^Kite Research Institute, Toronto Rehabilitation Institute, University Health Network, Toronto, ON, Canada; ^3^Japan Society for the Promotion of Science, Tokyo, Japan; ^4^Department of Life Sciences, Graduate School of Arts and Sciences, The University of Tokyo, Tokyo, Japan; ^5^Faculty of Sport Sciences, Waseda University, Tokyo, Japan

**Keywords:** corticospinal, neuroplasticity, motor point stimulation, transcranial magnetic stimulation, paired associative stimulation

## Abstract

Paired associative stimulation at the spinal cord (spinal PAS) has been shown to increase muscle force and dexterity by strengthening the corticomuscular connection, through spike timing dependent plasticity. Typically, transcranial magnetic stimulation (TMS) and transcutaneous peripheral nerve electrical stimulation (PNS) are often used in spinal PAS. PNS targets superficial nerve branches, by which the number of applicable muscles is limited. Alternatively, a muscle can be activated by positioning the stimulation electrode on the “motor point” (MPS), which is the most sensitive location of a muscle to electrical stimulation. Although this can increase the number of applicable muscles for spinal PAS, nobody has tested whether MPS can be used for the spinal PAS to date. Here we investigated the feasibility of using MPS instead of PNS for spinal PAS. Ten healthy male individuals (26.0 ± 3.5 yrs) received spinal PAS on two separate days with different stimulation timings expected to induce (1) facilitation of corticospinal excitability (REAL) or (2) no effect (CONTROL) on the soleus. The motor evoked potentials (MEP) response curve in the soleus was measured prior to the spinal PAS, immediately after (0 min) and at 10, 20, 30 min post-intervention as a measure of corticospinal excitability. The post-intervention MEP response curve areas were larger in the REAL condition than the CONTROL conditions. Further, the post-intervention MEP response curve areas were significantly larger than pre-intervention in the REAL condition but not in the CONTROL condition. We conclude that MPS can facilitate corticospinal excitability through spinal PAS.

## Introduction

Paired associative stimulation (PAS), first introduced by Stefan et al. (2000), utilizes repetitive and paired transcranial magnetic stimulation (TMS) on the motor cortex and peripheral nerve electrical stimulation (PNS) to the innervating nerves of a target muscle. By controlling the timing between these two stimuli, long-term potentiation (LTP) or long-term depression through spike-timing dependent plasticity (STDP) (Bi and Poo, 1998) can be induced. The majority aim to induce STDP at the cortical level (Stinear and Hornby, 2005; Prior and Stinear, 2006; Mrachacz-Kersting et al., 2007; Roy et al., 2007; Kumpulainen et al., 2012, 2015; Mrachacz-Kersting, 2013), while some aim at the spinal cord level (Taylor and Martin, 2009; Cortes et al., 2011; Bunday and Perez, 2012; Leukel et al., 2012; Shulga et al., 2016b; Knikou, 2017). Cortices are well-known to have an ability for neuroplasticity, but the spinal cord has it as well (Rossignol et al., 2007). PAS aiming at the spinal cord is often called spinal PAS (Shulga et al., 2015) or paired corticospinal-motoneuronal stimulation (PCMS) (Bunday et al., 2018). These investigations were first done by Taylor and Martin (2009) demonstrating similar LTP-like plasticity in healthy individuals, and repeated in individuals with spinal cord injury (SCI) by Bunday and Perez (2012).

Spinal PAS utilizes both TMS and PNS to induce STDP at the corticospinal-motoneuronal synapses via synchronized firing of upper and lower motor neurons. The upper motor neurons are activated via TMS while the lower motor neurons are activated by the antidromic propagation of PNS and orthodromic discharge from the descending TMS signal, with an interstimulus interval (ISI) for the two stimuli to collide at the corticospinal-motoneuronal synapses in the spinal cord. Spinal PAS increases the corticospinal-motoneuronal synapse excitability resulting in increasing muscle voluntary force and upper arm's dexterity in healthy individuals and those with SCI (Taylor and Martin, 2009; Roy et al., 2010; Bunday and Perez, 2012; Shulga et al., 2015; Fitzpatrick et al., 2016; Urbin et al., 2017; D'Amico et al., 2020). Furthermore, the effects can last for up to a month after a series of treatments in individuals with spinal cord injuries (Shulga et al., 2016a; Tolmacheva et al., 2017, 2019).

Current PAS protocols rely on PNS and are consequently limited to muscles where the innervating nerve is located superficially. This severely limits its application, as accessible muscles are limited. For instance, the nerve innervating the flexor and extensor muscles of the forearm may be relatively deep resulting in inconsistencies with stimulation if any movements may occur through the PAS protocol. Also, the nerves innervating the thigh muscles may also be located relatively deep below both muscles and fat tissue making it difficult to properly activate the nerve fibers. Additionally, the nerves activated by PNS may innervate other muscles leading to non-specific changes or interactions. An alternative to PNS for delivering peripheral electrical stimulation is motor point stimulation (MPS). For MPS, electrical stimulation is delivered above the motor point of the target muscle, which is a location most sensitive to electrical stimulation. MPS can induce antidromic discharge up the motor nerve (Nakagawa et al., 2020) which is a critical component of PAS protocols. Further, it is often used in clinical settings, where MPS is often called neuromuscular electrical stimulation or functional electrical stimulation (FES). Repetitive use of FES, called FES therapy, is known to induce “carry-over” effects. That is, individuals with neurological problems related to motor functions such as SCI and stroke can improve motor functions after performing FES therapy for weeks or months (Pomeroy et al., 2006; Ambrosini et al., 2011; Popovic et al., 2011b). These functional improvements are often accompanied by increases in motor evoked potential (MEP) (Everaert et al., 2010; Sugawara et al., 2014; Jochumsen et al., 2016). During FES therapy, participants are encouraged to participate through voluntary effort while MPS is applied. This can result in a similar situation to spinal PAS. Specifically, the voluntary descending command can meet antidromic firings from MPS at the spinal cord, which could lead to STDP (Rushton, 2003; Thompson and Stein, 2004; Popovic et al., 2011a).

To date no study has tried utilizing MPS in place of PNS in a spinal PAS protocol. Here we investigated the feasibility of using MPS instead of PNS for spinal PAS. We tested whether a spinal PAS protocol using MPS on healthy participants can induce STDP resulting in increases of corticospinal excitability measured using MEPs.

## Materials and Methods

### Participants

Ten male able-bodied participants (Age: 26.0 ± 3.5 years, mean age ± SD) with no known signs of neurological or musculoskeletal impairments participated in the experiment. All participants gave their written informed consent to participate in the study, whose experimental procedures were approved by the local ethics committee, and the study was conducted according to the Declaration of Helsinki.

### Intervention Protocol

**Figure 1:**
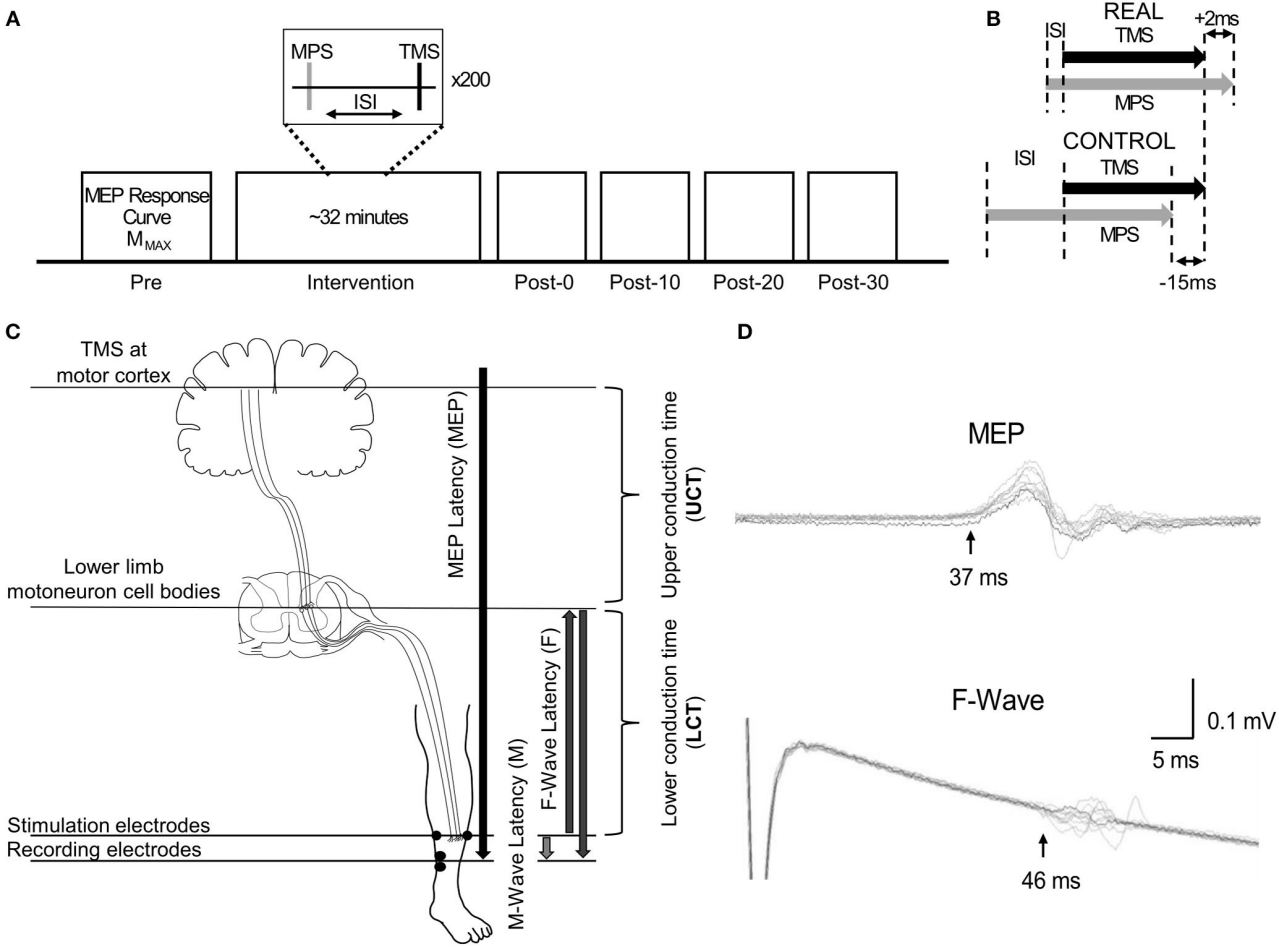
**(A)** Representation of the experimental protocol, highlighting the pre- and post-intervention assessment measurements. **(B)** Schematic descriptions of ISI for the REAL and CONTROL conditions. **(C)** Schematic description of the conduction time measurements done prior to PAS intervention. The abbreviations of each measurement are shown in the brackets. The MPS electrodes and the SOL EMG electrodes placement are also shown. **(D)** Time courses of measured EMGs showing MEPs and F-waves for a representative participant recorded from the SOL. Onset latencies are shown by the black vertical arrow with the corresponding MEP and F-wave latency for this participant.

Participants participated in two blinded experimental conditions separated by at least 24 h. The study consisted of a spinal PAS protocol like those used by Taylor and Martin (2009) and Bunday and Perez (2012) intended to facilitate corticospinal-motoneuronal synapse excitability (REAL condition) and a protocol expected to induce no change (CONTROL condition) on the medial soleus (SOL) muscle. The conditions were determined by two different ISIs. An ISI where the pre-synaptic signal arrived at the corticospinal-motoneuronal synapse 2 ms before the post-synaptic signal was used for the REAL condition. Conversely, an ISI where the pre-synaptic signal arrived at the corticospinal-motoneuronal synapse 15 ms after the post-synaptic signal was used for the CONTROL condition. Information on the calculation of these ISIs can be found in *Calculation of Interstimulus Intervals*. During testing participants were seated in an armchair with their left foot secured to a force transducer. For each experiment, participants were tested at rest and given 200 pairs of stimuli delivered at 0.1 Hz (32 min). While upper limb studies have shown consistent results with 100 pairs of stimuli (Taylor and Martin, 2009; Bunday and Perez, 2012), preliminary results were inconsistent using 100 pairs of stimuli. As some previous studies have used a minimum of 200 pairs of stimuli when targeting the lower limb (Shulga et al., 2015; Urbin et al., 2017), we also found that this made results more consistent compared to 100 pairs of stimuli in our pilot experiment agreeing with a previous study done by Fitzpatrick et al. (2016) demonstrating that more conditioning stimuli enhances the effect of PCMS. The TMS intensity was set at 120% of the resting motor threshold (RMT) of the SOL (Stefan et al., 2000) and the MPS was set to be 120% of M_MAX_ to maximize the activated motor neurons. This supra-maximum intensity is often used in PAS with PNS (Bunday et al., 2018). Throughout the intervention, the left foot remained attached to the foot plate to record twitch forces throughout the intervention. For this study, participants were instructed to stay awake and relax throughout. Figure 1 shows a schematic of the experimental protocol (Figure 1A), and the order of stimulation arrival at spinal synapses for each experiment (Figure 1B).

#### Target Muscle

The target muscle of the intervention protocol was the SOL. This study is a proof-of-concept study, but to enable comparison to the standard spinal PAS protocols using PNS in the future the SOL was chosen since the tibial nerve is accessible for PNS. Additionally, while several cortical-level (Stinear and Hornby, 2005; Prior and Stinear, 2006; Mrachacz-Kersting et al., 2007; Roy et al., 2007; Kumpulainen et al., 2012, 2015; Mrachacz-Kersting, 2013) and spinal level PAS (Roy et al., 2010; Cortes et al., 2011; Shulga et al., 2015; Urbin et al., 2017) studies targeting the lower limbs exist, the protocol used varies significantly making comparisons amongst the studies difficult. Consequently, lower limb PAS studies are still limited. Here we test the SOL to further study spinal PAS effects on the lower limbs and lay preliminary work to utilize spinal PAS to improve postural balance.

#### Electromyography, EMG

Electromyography (EMG) was recorded from the left SOL and tibialis anterior (TA) of all participants (MEG-6108, Nihon Kohden, Japan). Adhesive foam Ag/AgCl electrodes (Vitrode F-150S, Nihon Kohden, Japan) were placed in a bipolar configuration 1 cm apart on each muscle, and a common reference was placed on the right medial malleolus. The EMG signals were amplified and bandpass filtered from 1.5–1,000 Hz and sampled at 4 kHz for offline analysis.

#### Transcranial Magnetic Stimulation, TMS

A TMS system (Magstim 200, Whitland, United Kingdom) was used with a double cone coil positioned over the vertex and oriented to preferentially activate the right motor cortex. The hotspot was defined as the optimal position for eliciting a MEP in the SOL muscle with the minimum intensity (Christiansen et al., 2018). The location of the hotspot was saved/marked in the brain navigation software (Brainsight TMS Navigation system, Brainbox, Cardiff, United Kingdom). After identifying and saving the hotspot in the brain navigation system, the RMT was determined. The RMT was defined as the minimum intensity required to produce MEPs that are >50 μV peak-to-peak amplitude in 5 out of 10 consecutive trials when the muscle is relaxed (Rothwell et al., 1999; Christiansen et al., 2018). An intensity of 120%RMT was used during the intervention in both conditions. This protocol was executed at each visit. A summary of the stimulation thresholds is shown in Table 1.

**Table 1:** Summary of stimulation thresholds for TMS and MPS.

	**REAL**	**CONTROL**	***P*-values**
**Thresholds**			
RMT (%SO)	47.6 ± 6.2	48.0 ± 9.0	0.721
MT (mA)	74.2 ± 14.6	80.4 ± 11.7	0.215
**Response latencies**			
MEP (ms)	32.8 ± 2.1	32.8 ± 1.8	1.00
F-wave (ms)	41.0 ± 4.4	40.6 ± 4.7	0.509
ISI (ms)	6.2 ± 3.1	22.8 ± 3.4	<0.001

*Summary of MEP and F-wave latencies used to calculate individual ISI for each participant for each condition. Values are reported as mean ± SD. A paired t-test with a significance level of 0.05 was used to test for differences between the REAL and CONTROL condition values*.

#### Motor Point Stimulation, MPS

Among the ten participants, a constant current stimulation (1 ms pulse duration, model DS7AH, Digitimer, Welwyn Garden City, Unitd Kingdom) was used to deliver MPS to 5 participants, while a constant voltage stimulator (1 ms pulse duration, SEN-3301, Nihon-Kohoen, Tokyo, Japan) was used to deliver MPS to the remaining participants. The Digitimer malfunctioned after 5 participants prompting the change of stimulator devices. Since generating M_MAX_ is critical to ensure all neurons are activated, the constant voltage setting (100V limit) was tested and was able to achieve M_MAX_ in the participants. The observed effects of both stimulators were similar.

The motor point was searched via a handheld electrode and identified when the SOL was activated with the lowest motor threshold (Gobbo et al., 2014). The anode was placed on the lateral motor point while the cathode was placed on the mirrored motor point on the medial side location of the SOL, a depiction of the placement of the electrodes can be seen in Figure 1C. Painfulness assessments were not performed for participants and are thus not reported. For each experiment and participant, the stimulation intensity was increased until no increases were seen in SOL M-wave peak-to-peak amplitude and an intensity of 1.2 times above this intensity was used (Bunday and Perez, 2012; Bunday et al., 2018). This protocol was executed at each visit. A summary of the stimulation thresholds is shown in Table 1.

##### M_*MAX*_

The M_MAX_ of the SOL was recorded three times using MPS. An intensity of 1.2 times the intensity required to no longer see an increase in peak-to-peak amplitude was used. SOL M_MAX_ amplitude was recorded to rule out any changes in MEPs due to changes in muscle fiber action potentials. These measurements were taken prior to the intervention (pre), immediately after and at 10-, 20- and 30-min post intervention (Bunday and Perez, 2012).

#### Calculation of Interstimulus Interval

To calculate the interstimulus interval (ISI), the latencies of the SOL MEPs and F-waves were measured and used as described in previous spinal PAS studies (Shulga et al., 2015; Urbin et al., 2017). Figure 1C shows a schematic representation of the conduction pathways measured. The SOL MEP latency was measured by stimulating the hotspot at 120%RMT 10 times and the shortest latency among them was selected as the MEP latency (Figure 1D) (Groppa et al., 2012; Shulga et al., 2016b). F-waves were measured by electrically stimulating the SOL muscle motor point (i.e., MPS) at 120% M_MAX_ 10 times and the shortest latency was similarly taken (Figure 1D). Stimulation at supramaximal intensities on the motor point may activate the motor axons and subsequent proprioceptive afferent pathways. However, it has been shown that MPS activates the proprioceptive Ia afferent pathways less than PNS (Nakagawa et al., 2020). Thus, consideration of just the antidromic signal generated by MPS to calculate the ISI is appropriate since the antidromic signal arrives at the target synapse prior to reflexive actions. The onset latencies were defined as the onset of the response where the signal deviated from baseline (Shulga et al., 2015). A summary of the mean latencies is presented in Table 1. Once the SOL MEP latency and F-wave latencies were determined, the ISI was calculated as follows:

(1) $$\begin{array}{l} \text{LCT}=\;\frac{F\;-M}{2} \end{array}$$

(2) $$\begin{array}{l} \text{UCT}=\text{MEP }-\;\frac{F+M}{2} \end{array}$$

(3) $$\begin{array}{l} \text{Coincident Timing }=\text{LCT}-\text{UCT} \end{array}$$

(4) $$\begin{array}{l} \text{CT}=\;\frac{F-M}{2}-\left(MEP-\frac{F+M}{2}\right)\;\\ \text{CT}=F-MEP\\ \text{ISI}=\text{CT}-2\;\left(\text{REAL}\right)\text{ or CT}\\ \;+15\;\left(\text{CONTROL}\right) \end{array}$$

where *F, M* and MEP are the F-, M-, and MEP-latencies, respectively, and LCT and UCT represent the lower and upper conduction times, respectively.

### Corticospinal Excitability

The MEP response curve was recorded as follows. The MEP was measured at 100, 110, 120, and 130%RMT, an adaptation of Fujio et al. (2018), with 5 measurements per intensity delivered at 0.2 Hz (Roy et al., 2010). The order of the stimulation intensities were randomized. These measurements were taken prior to the intervention (pre), immediately after and at 10-, 20-, and 30-min post-intervention (Bunday and Perez, 2012).

All subsequent analyses were done using Matlab (MathWords Ltd., Nattick, MA, United States). The MEP response was quantified as the amplitude from peak to peak and five MEP amplitudes recorded were averaged when calculating the group data (Urbin et al., 2017). For the SOL MEPs, the averaged amplitudes were normalized to M_MAX_ at the same time assessment. While 5 consecutive MEP measurements are generally considered sufficient to assess MEP size (Groppa et al., 2012), it has also been recommended to record more MEP measurements to improve between-session reliability (Cavaleri et al., 2017). However, recording more MEP measurements has a drawback of requiring more time at each post-intervention assessment. Additionally, when observing each individual's averaged MEP amplitude response, they varied considerably. Consequently, instead of recording more MEP measurements, we calculated the area under the MEP response curve at each time point to incorporate in total 20 MEP measurements at each time assessment to improve both the between-session reliability and reduce the variability per participant. Changes in the corticospinal excitability were assessed by changes in this MEP response curve area (Carson et al., 2013; Potter-Baker et al., 2016). The MEP response curve area has been shown to be strongly related to maximum MEP amplitude (Talelli et al., 2008; Singh et al., 2014; Potter-Baker et al., 2016). Previous studies have successfully modulated low threshold motor neurons (Taylor and Martin, 2009; Fitzpatrick et al., 2016); however, higher threshold motor neurons have had varying levels of success (D'Amico et al., 2017; Dongés et al., 2019). Consequently, the effect of spinal PAS on corticospinal transmission to motor neurons may depend on the threshold of the neurons. As a result, using the MEP response curve area does not enable us to discern any differences since responses to individual stimulation intensities is lost. Conversely, by using the MEP response curve the number of analyzed data points per participant is increased which decreases the overall error. Results using individual TMS intensities is presented in Appendix A.

### Statistical Analysis

As one participant was found to have a SOL MEP amplitude >3 standard deviations from the group mean, we decided to omit this participant from subsequent analyses as an outlier. When assessing individual data, the 5 measurements per intensity were kept separate and used to calculate 5 areas under the MEP response curve. The normality of group and individual data was tested using the Shapiro–Wilk test. The individual data was found to be non-normal in 10% of the data across time assessments, conditions and muscles. The group data was found to be non-normal in 70% of the time assessments, conditions and muscles. A log base 10 (log10) transform was used on the data and retested for normality. After log transformation, the data was normally distributed. The sphericity was tested using the Mauchly's test.

A two-way repeated measures ANOVA with Holm–Bonferroni correction as a *post-hoc* was performed to determine the effect of time (pre, post-0, post-10, post-20, and post-30) and condition (REAL, CONTROL) on the log transformed MEP response curve area for the SOL and TA muscles separately for both the individual and group data. For the individual participant data, *post-hoc* evaluates were done via independent *t*-tests between post-intervention assessments and the pre-intervention assessments for each condition and muscle. *Post-hoc* evaluations for group data were done via paired *t*-tests between post-intervention assessments and the pre-intervention assessment for each condition and each muscle. Paired *t*-tests were used to examine differences in MEP and F-wave latencies, TMS intervention stimulation intensities, and MPS intervention stimulation intensity between the REAL and CONTROL conditions. Values were reported as mean ± standard deviation (SD). A statistical software (SPSS Statistics ver. 25, IBM Corp., United States) was used for all statistical tests. *p* <0.05 served as the significance level.

## Results

**Figure 2:**
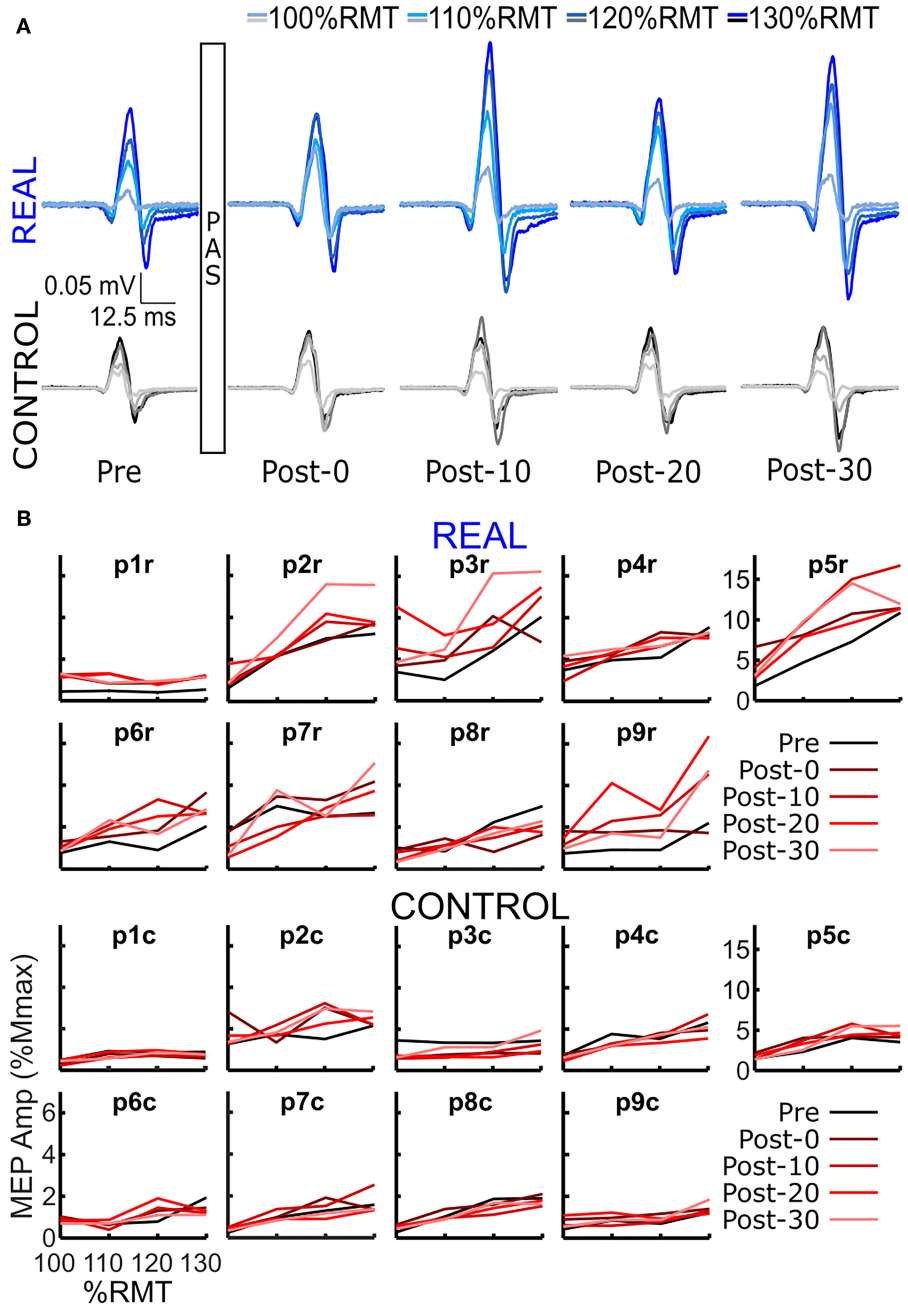
**(A)** SOL MEP traces in a representative participant before (pre-intervention) and after (post-intervention: post-0, post-10, post-20, post-30) the spinal PAS intervention for the REAL and CONTROL conditions. The traces represent the averages of 5 MEPs. There are four traces representing the four different TMS stimulation intensities used: 100%RMT, 110%RMT, 120%RMT, and 130%RMT in a light to dark gradient of blue/black for REAL and CONTROL conditions, respectively. **(B)** Individual SOL MEP response curves for all 9 participants in the REAL (top 2 rows) and CONTROL (bottom 2 rows) conditions.

SOL MEP traces of a representative participant for the REAL and CONTROL conditions are shown in Figure 2A. The amplitude of SOL MEP response increased from the pre-intervention assessment in the REAL condition PAS intervention, but not in the CONTROL condition. Figure 2B shows the individual SOL MEP response curves quantifying each peak-to-peak amplitude for the REAL (top two rows) and CONTROL (bottom 2 rows) conditions. One participant (p8r) was found to be a non-responder where potentiation of the average SOL MEP response curve area across post-intervention time assessments was <110% in the REAL condition (Bunday et al., 2018) and was excluded from further analysis.

**Figure 3:**
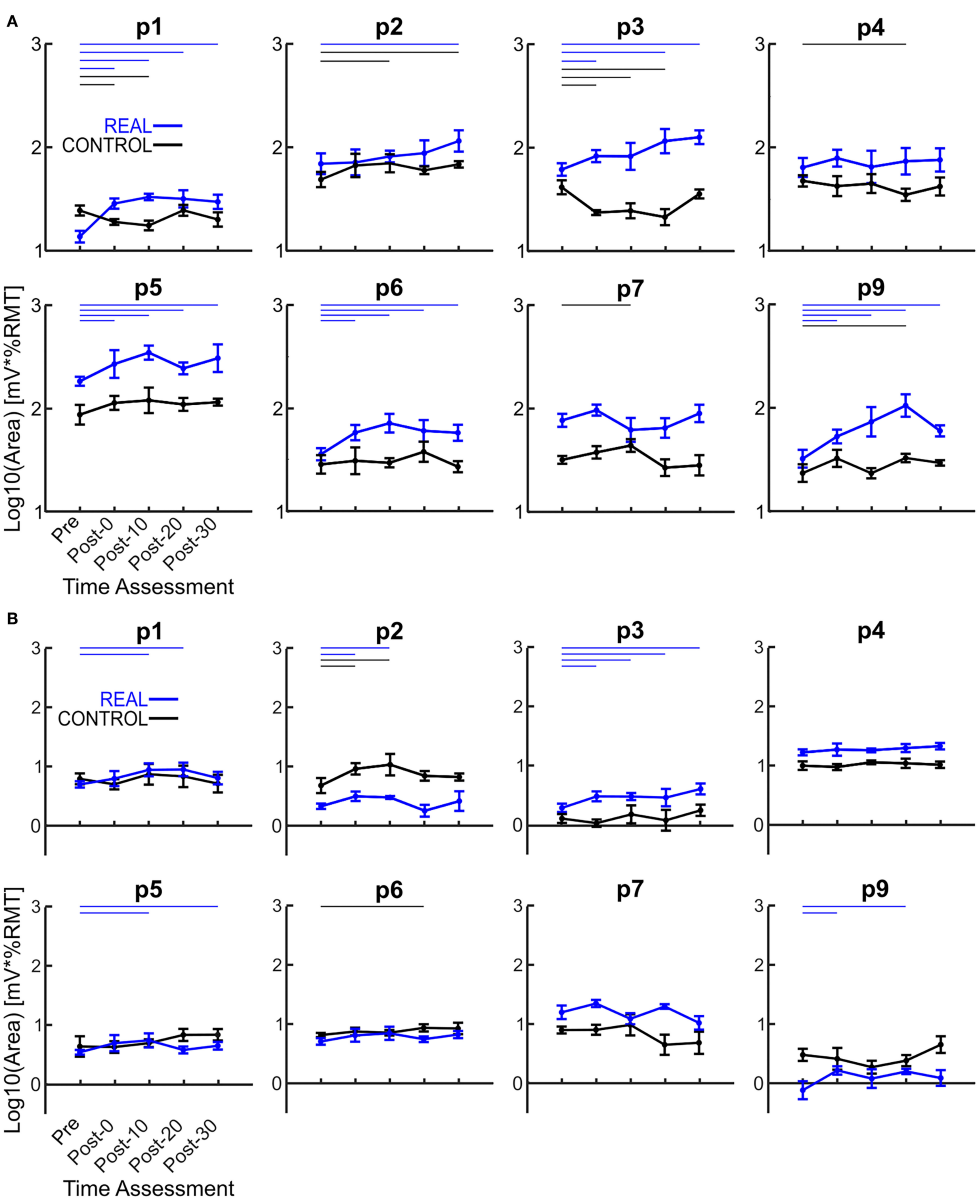
**(A)** Log transformed SOL MEP response curve areas for each participant. **(B)** Log transformed TA MEP response curve areas for each participant. Thick lines represent the group means for the REAL (blue) and CONTROL (black) conditions, vertical bars represent one standard deviation. Thin horizontal lines at the top of the graph denotes *p* < 0.05 when compared to the pre-intervention for the REAL (blue) or CONTROL (black) conditions.

The log transformed area under the SOL MEP response curves are shown in Figure 3A for each participant analyzed. A two-way repeated measures ANOVA was performed for each participant revealing a significant condition effect in all participants, a significant time effect in 6 participants, and a significant interaction effect in 5 participants. Independent *t*-tests with a Holm-Bonferroni correction revealed significant increases from the pre-intervention assessment for at least one post-intervention assessments in the REAL condition for 7 participants (Figure 3A). For the CONTROL condition, a significant difference from the pre-intervention assessment was found in at least one post-intervention assessment in 6 participants. For the TA, a two-way repeated measures ANOVA was performed on the log transformed TA MEP response curve areas for each participant. The two-way repeated measures ANOVA revealed a significant condition effect in 7 participants, a significant time effect in 6, and a significant interaction effect in 4 participants. *Post-hoc* independent *t*-tests with a Holm-Bonferroni correction revealed significant increases from the pre-intervention assessment in some post-intervention assessments for 5 participants in the REAL condition (Figure 3B). Significant increases from the pre-intervention was present at post-20 for one participant and at post-0 and post-10 for another participant in the CONTROL condition. Summary tables of all test statistics for individual participants including *p*-values and effect sizes (i.e., Hedges' G) can be found in Appendix B.

**Figure 4:**
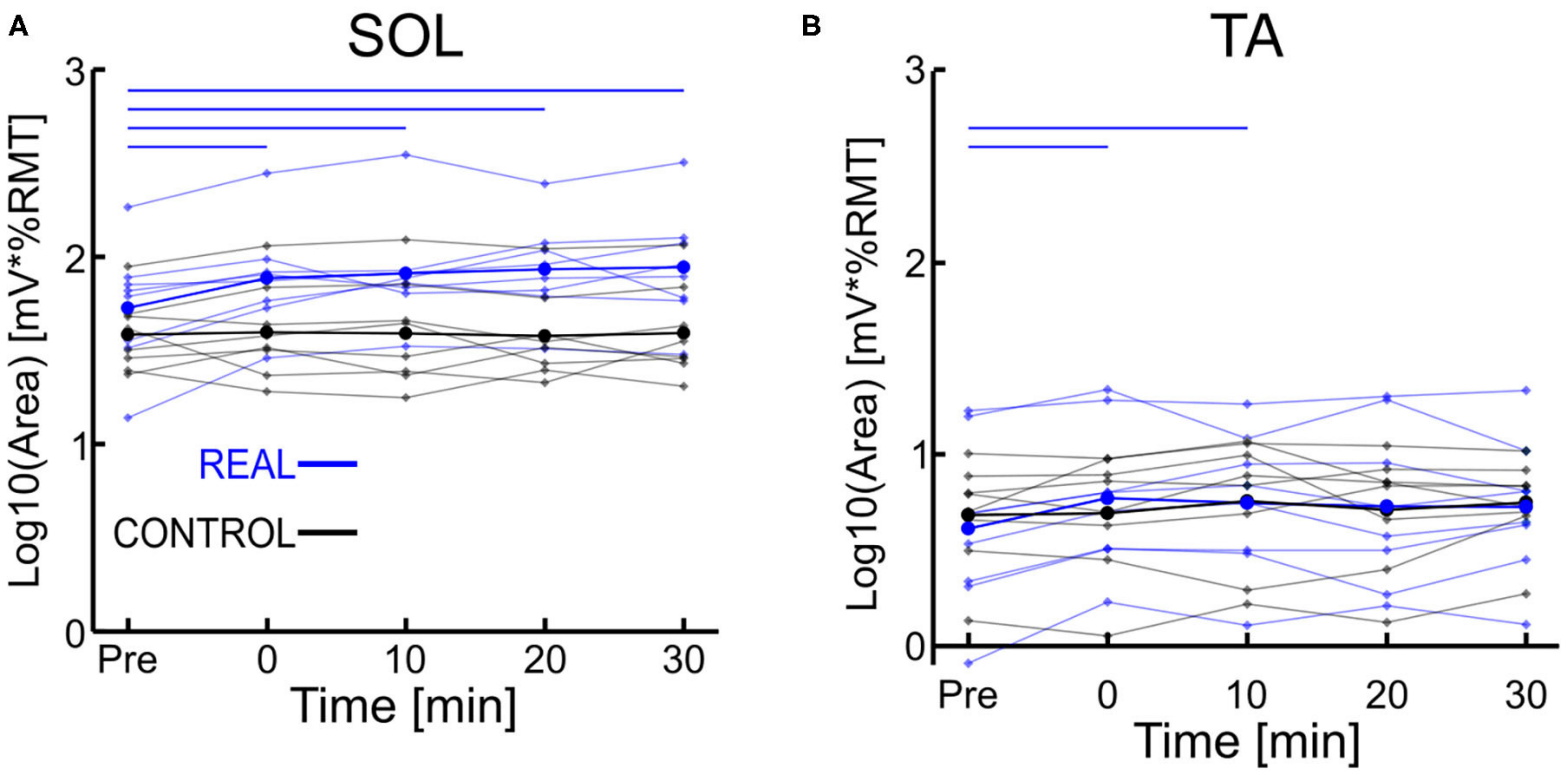
**(A)** Log transformed SOL MEP response curve area. **(B)** Log transformed TA MEP response curve area. Thick lines represent the group means for the REAL (blue) and CONTROL (black) conditions, while individual participants are shown as thin lines. Thin horizontal blue lines at the top of the graph denotes *p* < 0.05 when compared to the pre-intervention for the REAL condition.

Each log transformed area under the MEP response curve in Figure 3 was quantified and plotted in Figure 4A for SOL and Figure 4B for TA as the group results. For the SOL (Figure 4A), a two-way repeated measures ANOVA revealed a significant main effect of condition [*F*_(1, 7)_ = 35.838, *p* = 0.001, η^2^ = 0.837], a significant main effect of time [*F*_(4, 28)_ = 4.293, *p* = 0.008, η^2^ = 0.380] and a significant interaction effect [*F*_(4, 28)_ = 4.226, p = 0.008, η^2^ = 0.376] were found. Paired *t*-tests with a Holm–Bonferroni correction revealed significant increases from pre-intervention for all post-intervention assessments in the REAL condition, but no significant increases from pre-intervention were found in the CONTROL condition. The test statistic summary is shown in Table 2.

**Table 2:** Summary of the paired *t*-test statistics when comparing the post-intervention to pre-intervention assessment results for the log transformed SOL MEP response curve in both REAL and CONTROL condition.

**REAL**	**Post-0**	**Post-10**	**Post-20**	**Post-30**
t(7)	4.762	2.984	3.110	6.229
*P*-value	0.006	0.034	0.034	0.002
Geometric mean difference	1.438	1.531	1.605	1.649
95% CI	1.201–1.722	1.093–2.146	1.120–2.301	1.364–1.995
Hedges' G	0.467	0.543	0.635	0.623
**CONTROL**				
t(7)	0.274	0.137	−0.107	0.285
*P*-value	1.000	1.000	1.000	1.000
Geometric mean difference	1.031	1.016	0.987	1.021
95% CI	0.789–1.348	0.774–1.333	0.739–1.318	0.856–1.219
Hedges' G	0.055	0.026	0.024	0.038

For TA (Figure 4B), a two-way repeated measures ANOVA revealed no significant main effect of condition [*F*_(1, 7)_ < 0.001, *p* = 0.990, η^2^ = 0.000] or time [*F*_(4, 28)_ = 2.474, *p* = 0.067, η^2^ = 0.261]. Also, no significant interaction effect was found [*F*_(4, 28)_ = 1.448, *p* = 0.244, η^2^ = 0.171]. Paired *t*-tests with a Holm–Bonferroni correction revealed a significant increase from pre-intervention at post-0 and post-10 assessments after the REAL condition intervention, but not at other post-intervention assessments. The test statistic summary is shown in Table 3.

**Table 3:** Summary of the paired *t*-test statistics when comparing the post-intervention to pre-intervention assessment results for the log transformed TA MEP response curve in both REAL and CONTROL condition.

**REAL**	**Post-0**	**Post-10**	**Post-20**	**Post-30**
t(7)	5.747	3.174	2.612	2.302
*P*-value	0.003	0.047	0.070	0.070
Geometric mean difference	1.444	1.362	1.304	1.300
95% CI	1.241–1.679	1.082–1.714	1.026–1.658	0.993–1.701
Hedges' G	0.347	0.296	0.239	0.253
**CONTROL**				
t(7)	0.217	1.315	0.580	1.368
*P*-value	1.000	0.855	1.000	0.855
Geometric Mean difference	1.021	1.182	1.067	1.159
95% CI	0.814–1.281	0.875–1.596	0.820–1.388	0.898–1.497
Hedges' G	0.028	0.217	0.088	0.235

## Discussion

In spinal PAS, when the pre-synaptic volleys induced via TMS arrived 2 ms prior to the post-synaptic volleys from MPS, the SOL MEP response increased at the post-intervention assessments (Figures 2, 4A). Conversely, the SOL MEP response when the post-synaptic volleys arrived at the spinal cord 15 ms prior to the pre-synaptic volleys did not change (Figures 2, 4A). These results suggest spinal PAS protocols using MPS increased corticospinal excitability through STDP at the corticospinal synapse of the SOL in the spinal cord.

### PAS Using MPS

It is thought that PAS induces synapse-specific neuroplasticity, but for cortical-level PAS protocols such as Stefan et al.'s (2000) seminal work, stimulation of the median nerve to induce MEP increases in the abductor pollicus brevis resulted in simultaneous increases to the abductor digiti minimi and biceps brachii muscle to lesser degrees. Further upper limb studies using the same or similar protocols have demonstrated a similar non-specificity (Quartarone et al., 2003; Weise et al., 2006). While for lower limbs, a walking PAS protocol targeting the excitability of the TA increased SOL excitability as well (Stinear and Hornby, 2005). For cortical and spinal PAS protocols, the relevant nerves for PNS may innervate many other muscles leading to unintended interaction effects between muscles. Unfortunately, few lower limb TMS studies have recorded from more than two muscles simultaneously (Kesar et al., 2018). The literature for spinal PAS protocols on muscle specificity tends to record only from the single target muscle, as a result it is not known whether spinal PAS protocols are equally as non-specific. Also, stimulation of the sensory nerve fibers during PNS may decrease the tolerability of the stimulation, leading to reduced stimulation intensity and effectiveness (Reilly, 1992). An alternative to PNS is MPS, which focuses the stimulation up the motor neuron reducing transmission along the sensory nerve fibers of the mixed peripheral nerve resulting in improved comfort and tolerability (Gobbo et al., 2011, 2014). As a result, it is possible to use a stimulation intensity to fully activate all motor neurons without significant pain or discomfort. Consequently, this enables adequate post-synaptic activity of the spinal motor neurons to properly induce LTP-like effects. Further, given the similarity between FES therapy and PAS interventions, it is likely their effect on neuroplasticity are due to a similar mechanism. That is, the interaction between descending (TMS/voluntary commands) and ascending (PNS/MPS) signals induces neuroplastic changes in the CNS (Rushton, 2003; Thompson and Stein, 2004; Popovic et al., 2011a). Specifically, the success of FES therapy using electrical stimulation over the motor point suggests the use of MPS in PAS protocols to induce neuroplastic changes should be possible.

To our knowledge, our study is the first to use MPS in a spinal PAS protocol. Recently, Foysal and Baker (2019) facilitated corticospinal excitability to the hand muscles in healthy individuals, using MPS in a cortical PAS protocol. The authors induced changes to the corticospinal excitability of several hand muscles to varying degrees simultaneously. For our non-targeted TA muscle, a significant increase was found at both post-0 and post-10 assessments after the REAL condition PAS intervention but not at other post-intervention assessments. In the CONTROL condition slight but not significant increases are present (Figure 4B). These results are surprising given the electrical stimulation preferentially activates the SOL motor neurons and the coincidence of TMS volleys to the TA motor neurons are not well-timed. It was expected that the SOL would be well-facilitated, and the TA would not be affected by our spinal PAS protocol. One possibility for this result may be the co-activation of the TA during the PAS intervention due to the difficulty of targeting a single lower limb using TMS (Kesar et al., 2018) leading to facilitation of the TA at the cortical and/or spinal levels. This repeated activation of the TA motor hot spot may act as a very low frequency repetitive TMS protocol. While it is generally thought that low frequency repetitive TMS protocols (<1 Hz) result in inhibitory effects (Klomjai et al., 2015), studies have used a variety of parameters and produced some contradicting results (Fitzgerald et al., 2006). For instance, the effect on MEP size in low frequency repetitive TMS protocols has been found to be stimulation train length and intensity dependent (Fitzgerald et al., 2006). Indeed, recently D'Amico et al. (2020) demonstrated that repetitive TMS at low frequencies (0.1 Hz) increased MEP sizes. Their results showed increases in MEP sizes when a TMS stimulation intensity sufficient to induce MEP max was used, but not when an intensity of 110%RMT was used. Our current study utilizes a TMS intensity of 120%RMT, which would suggest no impact based on D'Amico's et al. recent results. However, they applied half the number of stimulations (100) than the current study (200). Given changes to MEP size are related to both stimulation intensity and duration (Maeda et al., 2000; Modugno et al., 2001; Fitzgerald et al., 2002) it is possible lower intensity, but longer durations produce similar effects to high intensity repetitive TMS at 0.1 Hz. Alternatively, a study by Foysal and Baker (2020) found that repetitive low frequency TMS at an intensity of 110%RMT coupled with motor imagery for 90 stimuli was sufficient to increase MEP sizes. This may suggest that while our subjects were instructed to relax throughout the intervention, there may have been some underlying activity in the motor cortex during the PAS intervention.

Overall, our TA MEP response curve area increases were slight suggesting a weak increase. Thus, the spinal PAS protocol using MPS may provide greater levels of specificity than spinal PAS protocols using PNS, but further work is required.

### Spike Timing Dependent Plasticity of the SOL

The SOL MEP response curve area for the REAL condition was found to have significant increases at all post-intervention assessment time points, but no such significant increases were found in the CONTROL condition. Further, for the post-intervention assessment in the REAL and CONTROL conditions, SOL MEP response curve areas were found to be significantly different from one another (Figure 4A). Previous studies using PNS have found significant increases in MEP amplitude immediately after PAS interventions and lasting at least until 30 min after (Bunday and Perez, 2012; Fitzpatrick et al., 2016; Urbin et al., 2017). Furthermore, two upper limb studies (Kujirai et al., 2006; Bunday et al., 2018) and one lower limb study (Mrachacz-Kersting et al., 2007) demonstrated that PAS performed during voluntary activity increased corticospinal excitability. An explanation for this may be the generation of many volleys prior to and after the intended collision of the TMS and MPS, which could be critical to the potentiation of the corticospinal synapses. Indeed, studies that utilize a train of peripheral stimulation instead of single pulses has shown an ability to facilitate MEP size over a wider range of ISIs which is a critical consideration when translating this intervention into clinical practice (Shulga et al., 2015, 2016b). Despite the sub-optimal method of observing PAS effects on the corticospinal excitability of the lower limbs, our results for our target SOL muscle are in line with the results of previous PAS studies. However, future experiments should consider the utilization of voluntary activation to further elucidate our post-intervention effects, while also increasing the post-intervention assessments upwards to 60 min to better observe the length of the effect.

For the CONTROL condition, we found no significant changes in the SOL MEP response curve area (Figure 4A, black color). Urbin et al. (2017) found that in their CONTROL condition (termed PCMS-), their healthy participants had reduced corticospinal excitability via significant decreases in MEP size for at least 30 min after the intervention. However, they also found that among their healthy participants there were two groups, one such group were responders (i.e., reduced corticospinal excitability) and the other were non-responders (i.e., no change in corticospinal excitability). These groups were about evenly sized (7 to 6, respectively), and the authors were unable to determine the mechanism for the cause of their non-responder group. Additionally, it has been shown that the electrical stimulation of homonymous and heteronymous nerves of the legs can facilitate or inhibit MEP sizes (Roy and Gorassini, 2008). More specifically, when electrical stimulation is delivered at the posterior tibial nerve 24–30 ms prior to delivering TMS to the SOL hotspot, significant MEP depressions can be observed. The average ISI used was about 23 ms (18–28 ms), however our modality of our electrical stimulation was MPS and located further anatomically from the posterior tibial nerve stimulation location. Thus, the timing range may not be within the 24–30 ms window to suppress MEP size. Thus, the results of our CONTROL condition may be due to our ISI range and difference in stimulation modality.

### Limitations

One limitation of the current study may be the sample size of 8 participants analyzed. Ideally, upwards of 15–20 participants would have been recruited, however this was not possible due to technical and time limitations of the exchange collaborative project. Another limitation is the lack of comparison to a spinal PAS protocol using PNS, thus it is not possible to compare the efficacy or similarities of spinal PAS using the two different forms of electrical stimulation. This is a next step that should be performed to further our understanding of spinal PAS interventions.

### Conclusions

In conclusion, we have demonstrated that paired stimuli from TMS and MPS carefully timed to arrive at the pre-synaptic and post-synaptic terminal 2 ms apart facilitates specific corticospinal excitability. While functional measures such as voluntary force were not assessed in this proof of concept study these results may provide an opportunity to individuals with SCI to unlock further therapeutic options. Ultimately, providing more rehabilitation opportunities than the intervention itself.

## Data Availability Statement

The raw data supporting the conclusions of this article will be made available by the authors, without undue reservation.

## Ethics Statement

The studies involving human participants were reviewed and approved by the Research Ethics Board at University Health Network. The patients/participants provided their written informed consent to participate in this study.

## Author Contributions

KF, NK, AS, KeN, KiN, and KM: conception and design of the study. KF, NK, and AS: acquisition of data. KF and KM: interpretation of the results. KF: drafting of the manuscript. KF, NK, AS, KeN, KiN and KM: revising the manuscript and final approval of the version to be published. All authors contributed to the article and approved the submitted version.

## Conflict of Interest

The authors declare that the research was conducted in the absence of any commercial or financial relationships that could be construed as a potential conflict of interest.
