# Supplementary material for: Motor Point Stimulation in Spinal Paired Associative Stimulation can Facilitate Spinal Cord Excitability
Source: Front Hum Neurosci. 2020 Nov 27;14:593806. doi: 10.3389/fnhum.2020.593806 (PMC7729006; doi:10.3389/fnhum.2020.593806)
Supplement: Supplementary file 1 [file Data_Sheet_1.pdf]

1 Appendix A – Group results for individual TMS stimulation intensity

For each intensity, the 5 peak-to-peak MEP amplitudes were averaged at each post-intervention assessment. The normality of the group data for each stimulation intensity and condition was tested using the Shapiro-Wilk test and was found to be non-normal. The log-transformation of this group data was found to be normal for each stimulation intensity and condition. Sphericity was tested using the Mauchly's test, and if sphericity could not be assumed a Greenhouse-Geisser correction was used. A two-way repeated measures ANOVA with Holm-Bonferroni correction as a post-hoc was performed to determine the effect of time (pre, post-0, post-10, post-20 and post-30) and condition (REAL, CONTROL) on the log transformed MEP amplitudes for the SOL muscle at each stimulation intensity for the group data. Post-hoc evaluations were done via paired t-tests between post-intervention assessments and the pre-intervention assessment for each condition and intensity.

The log transformed MEP amplitude for each stimulation intensity is shown in Figure A1 as the group results. For 100%RMT (Figure A1A), a two-way repeated measures ANOVA revealed a significant main effect of condition ($F(1,7) = 9.204$, $p = 0.019$, $\eta^2 = 0.568$), a significant main effect of time ($F(4, 28) = 3.409$, $p = 0.022$, $\eta^2 = 0.328$), but no significant interaction effect ($F(4,28) = 0.956$, $p = 0.405$, $\eta^2 = 0.120$) were found. Paired t-tests with a Holm-Bonferroni correction revealed a significant increase from pre-intervention immediately post-intervention in the REAL condition, but no significant increases from pre-intervention were found at any other post-intervention assessments in the REAL or in the CONTROL condition. The test statistic summary is shown in Table A1.

The log transformed MEP amplitude for each stimulation intensity is shown in Figure A1 as the group results. For 100%RMT (Figure A1A), a two-way repeated measures ANOVA revealed a significant main effect of condition ($F(1,7) = 38.380$, $p < 0.001$, $\eta^2 = 0.846$), no significant main effect of time ($F(4, 28) = 1.805$, $p = 0.156$, $\eta^2 = 0.205$), and no significant interaction effect ($F(1.537,10.762) = 3.681$, $p = 0.069$, $\eta^2 = 0.345$) were found. Paired t-tests with a Holm-Bonferroni correction revealed a significant increase from pre-intervention at Post-0 and Post-30 assessments in the REAL condition, but no significant increases from pre-intervention values were found at any other post-intervention assessments in the REAL or in the CONTROL condition. The test statistic summary is shown in Table A2.

The log transformed MEP amplitude for each stimulation intensity is shown in Figure A1 as the group results. For 100%RMT (Figure A1A), a two-way repeated measures ANOVA revealed a significant main effect of condition ($F(1,7) = 17.091$, $p = 0.004$, $\eta^2 = 0.709$), a significant main effect of time ($F(1.981, 12.868) = 4.905$, $p = 0.025$, $\eta^2 = 0.412$), and a significant interaction effect ($F(4,28) = 2.932$, $p = 0.038$, $\eta^2 = 0.295$) were found. Paired t-tests with a Holm-Bonferroni correction revealed a significant increase from pre-intervention at all post-intervention

assessments in the REAL condition, but no significant increases from pre-intervention values were found in the CONTROL condition. The test statistic summary is shown in Table A3.

The log transformed MEP amplitude for each stimulation intensity is shown in Figure A1 as the group results. For 100%RMT (Figure A1A), a two-way repeated measures ANOVA revealed a significant main effect of condition ($F(1,7) = 40.667$, $p < 0.001$, $\eta^2 = 0.853$), a significant main effect of time ($F(4, 28) = 3.452$, $p = 0.021$, $\eta^2 = 0.330$), but no significant interaction effect ($F(4,28) = 2.165$, $p = 0.099$, $\eta^2 = 0.236$) were found. Paired t-tests with a Holm-Bonferroni correction revealed a significant increase from pre-intervention at the Post-30 assessments in the REAL condition, but no significant increases from pre-intervention values were found at any other post-intervention assessments in the REAL or in the CONTROL condition. The test statistic summary is shown in Table A4.

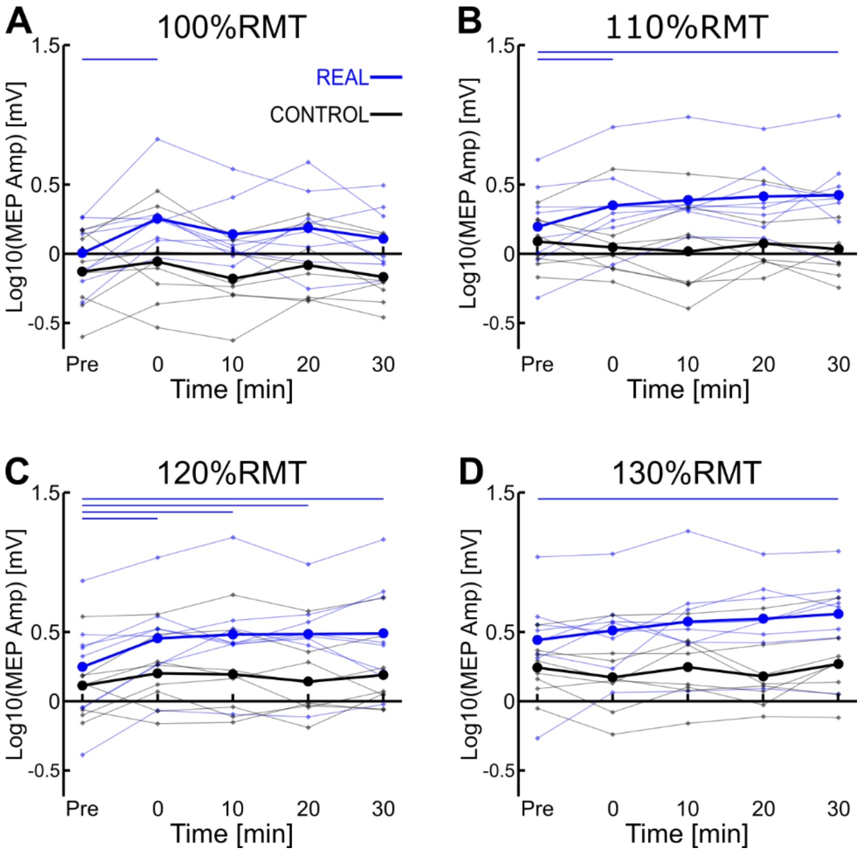

Figure A1 – Graphs show group data as thick lines for the REAL (blue) and CONTROL (grey) conditions when stimulated at 100%,110%,120%, and 130%RMT respectively, n = 8. Individual participants are shown as thin lines. Horizontal blue lines at the top of the graphs indicate a statistical difference from the pre-intervention MEP amplitude. (A) 100%RMT (B) 110%RMT (C) 120%RMT (D) 130%RMT.

Table A1 – Summary of the paired t-test statistics when comparing the post-intervention to pre-intervention assessment results for the log transformed SOL MEP amplitude in both REAL and CONTROL condition at 100%RMT.

REAL	Post-0	Post-10	Post-20	Post-30
t(7)	3.516	1.602	1.557	1.138
P-value	0.039	0.460	0.460	0.327
Geometric Mean difference	1.771	1.366	1.517	1.271
95% CI	1.206 - 2.600	0.862 - 2.163	0.805 - 2.858	0.772 - 2.090
Hedges' G	0.919	0.517	0.631	0.399
CONTROL				
t(7)	0.793	-0.603	0.564	-0.707
P-value	1.000	1.000	1.000	1.000
Geometric Mean difference	1.181	0.892	1.113	0.915
95% CI	0.718 - 1.943	0.570 - 1.396	0.710 - 1.746	0.679 - 1.232
Hedges' G	0.214	0.169	0.160	0.142

Table A2 – Summary of the paired t-test statistics when comparing the post-intervention to pre-intervention assessment results for the log transformed SOL MEP amplitude in both REAL and CONTROL condition at 110%RMT.

REAL	Post-0	Post-10	Post-20	Post-30
t(7)	3.732	2.534	2.031	6.207
P-value	0.022	0.078	0.082	0.002
Geometric Mean difference	1.425	1.561	1.653	1.693
95% CI	1.139 - 1.784	1.030 - 2.366	0.921 - 2.967	1.385 - 2.069
Hedges' G	0.460	0.613	0.691	0.669
CONTROL				
t(7)	-0.773	-1.023	-0.243	-1.657
P-value	1.000	1.000	0.930	0.566
Geometric Mean difference	0.910	0.849	0.968	0.880

95% CI	0.683 - 1.214	0.582 - 1.240	0.708 - 1.324	0.734 - 1.056
Hedges' G	0.169	0.247	0.062	0.247

Table A3 – Summary of the paired t-test statistics when comparing the post-intervention to pre-intervention assessment results for the log transformed SOL MEP amplitude in both REAL and CONTROL condition at 120%RMT.

REAL	Post-0	Post-10	Post-20	Post-30
t(7)	5.017	3.163	4.245	5.124
P-value	0.005	0.016	0.008	0.005
Geometric Mean difference	1.605	1.712	1.727	1.743
95% CI	1.284 - 2.006	1.145 - 2.558	1.274 - 2.341	1.349 - 2.253
Hedges' G	0.525	0.572	0.619	0.561
CONTROL				
t(7)	1.435	1.355	0.378	1.801
P-value	0.583	0.583	0.716	0.459
Geometric Mean difference	1.225	1.204	1.068	1.191
95% CI	0.877 - 1.713	0.871 - 1.663	0.708 - 1.611	0.947 - 1.497
Hedges' G	0.314	0.261	0.101	0.261

Table A4 – Summary of the paired t-test statistics when comparing the post-intervention to pre-intervention assessment results for the log transformed SOL MEP amplitude in both REAL and CONTROL condition at 130%RMT.

REAL	Post-0	Post-10	Post-20	Post-30
t(7)	1.095	2.656	2.469	4.059
P-value	0.310	0.098	0.098	0.019
Geometric Mean difference	1.170	1.353	1.422	1.542
95% CI	0.834 - 1.640	1.034 - 1.771	1.015 - 1.992	1.198 - 1.985
Hedges' G	0.187	0.343	0.424	0.513

CONTROL				
t(7)	-1.768	0.053	-1.521	0.488
P-value	0.482	1.000	0.516	1.000
Geometric Mean difference	0.851	1.005	0.865	1.063
95% CI	0.686 - 1.056	0.786 - 1.286	0.691 - 1.084	0.791 - 1.427
Hedges' G	0.285	0.010	0.262	0.107

2 Appendix B – Individual participant statistical test results for the MEP response curve area

Table B1 – Summary of the two-way repeated measures ANOVA on the log transformed SOL MEP response curve areas for each participant. Bolded values indicate p < 0.05.

Participant		df	F-value	p-value	η^2
	Condition	1	41.060	**0.003**	0.911
p1	Time	4	26.999	**<0.001**	0.871
	Interaction	4	48.344	**<0.001**	0.924
	Condition	1	29.615	**0.006**	0.881
p2	Time	4	4.380	**0.014**	0.523
	Interaction	4	2.194	0.116	0.354
	Condition	1	487.393	**<0.001**	0.992
p3	Time	4	15.096	**<0.001**	0.791
	Interaction	4	46.521	**<0.001**	0.921
	Condition	1	52.097	**0.002**	0.929
p4	Time	4	0.816	0.534	0.169
	Interaction	4	1.726	0.194	0.301
	Condition	1	134.422	<0.001	0.971
p5	Time	4	16.031	<0.001	0.800
	Interaction	1.207	1.398	0.304	0.259
	Condition	1	448.080	**<0.001**	0.991
p6	Time	4	10.333	**<0.001**	0.721
	Interaction	4	3.693	**0.026**	0.480
	Condition	1	388.095	**<0.001**	0.988
p7	Time	1.354	5.383	0.059	0.574
	Interaction	2.05	6.215	**0.022**	0.608
	Condition	1	168.959	**<0.001**	0.977
p9	Time	4	22.418	**<0.001**	0.849
	Interaction	4	11.410	**<0.001**	0.740

Table B2 – Summary of the independent t-test p-values and effect size (Hedges' G) when comparing the post-intervention to pre-intervention assessment results for the log transformed SOL MEP response curve in both the REAL and CONTROL condition. Bolded values represent significant differences (p < 0.05).

REAL	Post-0		Post-10		Post-20		Post-30	
	p-value	Hedges' G	p-value	Hedges' G	p-value	Hedges' G	p-value	Hedges' G
p1[#]	**<0.001**	5.473	**<0.001**	7.958	**<0.001**	4.743	**<0.001**	4.856
p2[#]	0.858	0.106	0.560	0.812	0.560	0.829	0.036	1.957

p3[#]	**0.018**	1.962	0.084	1.200	**0.005**	2.787	**<0.001**	4.440
p4[#]	0.565	0.935	0.947	0.041	0.880	0.492	0.880	0.646
p5[^]	**0.029**	1.699	**<0.001**	4.484	**0.014**	2.247	**0.015**	2.279
p6[^]	**0.003**	2.853	**0.001**	3.653	**0.003**	2.523	**0.003**	2.758
p7[^]	0.114	1.528	0.462	0.938	0.462	0.852	0.364	0.820
p9[^]	**0.003**	2.554	**0.003**	2.826	**0.000**	4.782	**0.001**	3.482
CONTROL								
p1[#]	**0.006**	2.635	**0.005**	2.769	0.955	0.033	0.099	1.340
p2[^]	0.082	1.317	**0.045**	1.768	0.082	1.465	**0.013**	2.554
p3[^]	**0.000**	4.864	**0.002**	2.936	**0.001**	3.604	0.108	1.057
p4[#]	0.821	0.601	0.681	0.324	**0.023**	2.135	0.821	0.690
p5[#]	0.183	1.262	0.183	1.146	0.163	1.135	0.113	1.697
p6[#]	1.000	0.288	1.000	0.209	0.289	1.184	1.000	0.286
p7[#]	0.154	1.342	**0.011**	2.516	0.186	1.159	0.290	0.708
p9[^]	0.084	1.530	0.975	0.019	**0.035**	2.093	0.084	1.593

[^] Indicates the participant received constant current stimulation via Digitimer. [#]Indicates the participant received constant voltage stimulation via Nihon-Kohoen.

Table B3 - Summary of the two-way repeated measures ANOVA on the log transformed TA MEP response curve areas for each participant. Bolded values indicate $p < 0.05$.

Participant		df	F-value	p-value	η^2
	Condition	1	4.464	0.102	0.527
p1	Time	4	8.865	**0.001**	0.689
	Interaction	4	1.687	0.202	0.297
	Condition	1	177.323	**<0.001**	0.978
p3	Time	4	12.895	**<0.001**	0.763
	Interaction	4	2.067	0.133	0.341
	Condition	1	79.560	**0.001**	0.952
p4	Time	4	6.712	**0.002**	0.627
	Interaction	4	4.196	**0.016**	0.512
	Condition	1	138.879	**<0.001**	0.972
p4	Time	4	1.807	0.177	0.311
	Interaction	4	1.661	0.208	0.293
	Condition	1	9.721	**0.036**	0.708
p5	Time	4	2.613	0.075	0.395
	Interaction	4	6.435	**0.003**	0.617
	Condition	1	10.707	**0.031**	0.728
p6	Time	4	7.095	**0.002**	0.639
	Interaction	4	2.070	0.133	0.341
	Condition	1	98.611	**0.001**	0.961
p7	Time	4	7.562	**0.001**	0.654

	Interaction	4	6.532	**0.003**	0.620
	Condition	1	71.994	**0.001**	0.947
p9	Time	4	5.885	**0.004**	0.595
	Interaction	4	8.123	**0.001**	0.670

Table B4 – Summary of the independent t-test p-values and effect size (Hedges' G) when comparing the post-intervention to pre-intervention assessment results for the log transformed TA MEP response curve in both the REAL and CONTROL condition. Bolded values represent significant differences ($p < 0.05$).

REAL	Post-0		Post-10		Post-20		Post-30	
	p-value	Hedges' G	p-value	Hedges' G	p-value	Hedges' G	p-value	Hedges' G
p1#	0.156	0.978	**0.008**	2.724	**0.008**	2.709	0.156	1.213
p2#	**0.010**	2.420	**0.001**	3.754	0.330	0.931	0.330	0.747
p3#	**0.008**	2.267	**0.005**	2.646	**0.044**	1.444	**0.001**	3.524
p4#	0.458	0.549	0.458	0.758	0.278	1.101	0.056	1.791
p5^	0.081	1.592	**0.030**	2.274	0.250	0.721	**0.034**	1.919
p6*	0.187	1.144	0.116	1.490	0.283	0.660	0.051	1.822
p7*	0.127	1.554	0.211	0.943	0.211	1.162	0.127	1.410
p9*	**0.008**	2.707	0.104	1.141	**0.008**	2.915	0.104	1.304
CONTROL								
p1#	0.563	0.935	1.000	0.519	0.822	0.281	1.000	0.604
p2^	**0.017**	2.272	**0.022**	2.064	0.085	1.410	0.085	1.381
p3^	0.346	1.014	0.753	0.564	0.753	0.213	0.137	1.467
p4#	1.000	0.333	0.592	0.978	1.000	0.477	1.000	0.225
p5#	1.000	0.068	1.000	0.420	0.223	1.285	0.223	1.327
p6#	0.230	1.052	0.230	0.828	**0.021**	2.245	0.137	1.490
p7#	0.935	0.049	0.663	0.662	0.061	1.959	0.122	1.576
p9^	0.501	0.418	0.059	1.769	0.317	0.889	0.173	1.280

^ Indicates the participant received constant current stimulation via Digitimer. # Indicates the participant received constant voltage stimulation via Nihon-Kohoen.
